# Subjective and objective cervical spine mobility after two-level anterior cervical discectomy and fusion - A prospective observational trial

**DOI:** 10.1016/j.bas.2026.106250

**Published:** 2026-07-30

**Authors:** M. Hohenhaus, M. Bissolo, S. Behringer, F. Volz, R. Watzlawick, J. Beck, J.-H. Klingler, U. Hubbe, C. Scholz

**Affiliations:** Department of Neurosurgery, Medical Center - University of Freiburg, Faculty of Medicine, University of Freiburg, Freiburg, Germany

**Keywords:** Anterior cervical discectomy and fusion, ACDF, Spinal stenosis, Degenerative cervical myelopathy, Cervical range of motion, Cervical mobility

## Abstract

**Introduction:**

Cervical spine mobility is an important functional outcome following surgical procedures. For single-level ACDF, several studies indicated that cervical range-of-motion is not compromised and may even improve, despite fusion of the treated segment.

**Research question:**

This study investigates the cervical mobility for two-level ACDF.

**Material and methods:**

Prospective, observational trial on patients prior, 3 months and 1 year after degenerative two-level ACDF concerning objective goniometric and subjective quality of their cervical range-of-motion. Pain courses for neck and arm, quality of life (SF-8, ADLs) and patient satisfaction were queried.

**Results:**

69 patients were analyzed (56.7 ± 11.0 years, 66.7% male). Goniometric range-of-motion worsened 3 months postoperatively in flexion-extension −11.5° (p < 0.001), lateral tilt −8.0° (p < 0.001) and rotation −3.6° (p = 0.062), regaining similar mobility to preoperative after 1 year (−1.5°, p = 0.700; −0.7°, p = 0.700; +2.9°, p = 1.000). Subjective range-of-motion improved significant after 3 months, and this improvement persisted even after 1 year. There was significant pain reduction at rest (NRS neck −1.2, p = 0.007; arm −2.0, p < 0.001) and under strain (NRS neck −1.5, p < 0.001; arm −2.9, p < 0.001) already after 3 months. Physical (+8.6, p < 0.001) and mental (+7.7, p = 0.065) component of SF-8 was improved and 92.8% were satisfied with the surgery.

**Conclusion:**

Two-level ACDF was not associated with deterioration in cervical spine mobility. Objective range-of-motion was temporarily reduced, returning to baseline after 1 year. Subjective mobility was already improved 3 months after surgery, accompanied by sustained pain reduction and improved quality of life. Consequently, concerns about postoperative motion reduction after two-level ACDF can be actively addressed.

## Highlights

• This is a prospective observation of the cervical spine mobility for two-level ACDF.
• Goniometric range-of-motion was temporarily reduced, but recovered after 1 year.
• Patient-perceived range-of-motion improved already significantly after 3 months.
• Significant enhanced quality of life and 92.8% satisfied patients after surgery.
• Concerns about motion restrictions after two-level ACDF can be actively addressed.

## Abbreviations

ACDF: Anterior Cervical Discectomy and Fusion
ADLs: Activities of Daily Living
BMI: Body-Mass-Index
CROM: Cervical Range-Of-Motion
DCM: Degenerative Cervical Myelopathy
MCS: Mental Component Summary
NRS: Numeric Rating Scale
PCS: Physical Component Summary
PROM: Patient-Reported Outcomes Measures
PRRS: Patient-Reported Restriction Score
PSI: Patient Satisfaction Index
HR-QoL: Health Related - Quality of Life
SF-8: Short Form Health Survey 8

## 1 Introduction

The mobility of the cervical spine represents an important functional outcome parameter after surgical interventions, as it directly influences activities of daily living (ADLs), occupational performance, and health-related quality of life (HR-QoL) (Langenfeld et al., 2018; Riffitts et al., 2023). In patients undergoing anterior cervical discectomy and fusion (ACDF), one of the most common interventions for the degenerative cervical spine, concerns regarding postoperative loss of motion are particularly common, given that the procedure intentionally eliminates mobility at the treated segment (Smith and Robinson, 1958; Hilibrand et al., 2006). Basically, this depends especially on the degree of cervical degeneration and the number of segments surgically addressed (Dvir et al., 2006; Simpson et al., 2008; Bell et al., 2011; Wu et al., 2012; Kasliwal et al., 2016)**.**

However, the relationship between segmental fusion and global cervical spine mobility is more complex than a simple reduction in cervical range-of-motion (CROM) after fixation of a segment. For single-level ACDF, several clinical and biomechanical studies demonstrated that the procedure does neither result in a clinically relevant nor objective deterioration of the global cervical mobility (Hilibrand et al., 2006; Landers et al., 2013; Scholz et al., 2021). Radiographic CROM measurements and goniometric analyses consistently showed, that all planes of motion are largely preserved after single-level surgery, or even improved. These effects seem to be related to the restoration of movement patterns once pain-induced guarding and protective muscle tension are alleviated (Scholz et al., 2021). Beside those objective assessments, patient-reported outcomes measures (PROM) indicated an increase in subjective cervical mobility following single-level ACDF (Scholz et al., 2021). Multiple studies reported improved patient-perceived CROM, neck function, and ease of performing ADLs, not isolated based on the pronounced analgesic effect of the surgery (Langenfeld et al., 2018; Riffitts et al., 2023; Scholz et al., 2021). Biomechanical studies showed that adjacent segments can partially compensate for the loss of motion at the fused level, while this compensatory hypermobility has been discussed as potential risk factor for adjacent segment degeneration in the long term, whereas short-to mid-term studies generally reported favorable functional outcomes with preserved mobility (Landers et al., 2013; Ishihara et al., 2004).

In summary, current evidence indicates that single-level ACDF does not adversely affect cervical mobility. These findings are highly relevant for preoperative patient counseling, as they challenge the intuitive assumption that spinal fusion inevitably results in functional stiffness. For patients with two-level surgeries, such evaluations are scarce and the effects on subjective cervical mobility, HR-QoL and ADLs were not addressed until now (Bell et al., 2011; Chien et al., 2015).

The aim of this study was to prospectively evaluate the impact of two-level ACDF on objective and subjective CROM, and to determine whether the effects observed after single-level interventions persist or disappear.

## 2 Materials and Methods

This was a prospective, single-center, observational trial on patients receiving two-level ACDF in a tertiary neurosurgical center between 12/2016 and 12/2019, whereas five patients were additionally included in 2022. The temporary suspension of admissions was due to COVID-19 pandemic. The trial was approved by the local ethics committee (EK 485/16) and registered in the National Clinical Trials Registry (DRKS00011277). All patients gave their written informed consent. Patients with traumatic, infectious or neoplastic pathologies were not included, resulting in an isolated degenerative cohort showing symptoms of radiculopathy or degenerative cervical myelopathy (DCM). Patients with a pacemaker had to be excluded, because of the strong magnet of the goniometer. The ACDF procedure was performed via an anterior right-sided approach and implantation of titanium intervertebral cages. No immobilization with neck braces was recommended after surgery.

The study-associated examinations were applied as shown by Scholz et al. for single-level ACDF (Scholz et al., 2021). After inclusion, patients received a standardized questionnaire for neck and arm pain at rest and under strain as well as under each plane of movement of the cervical spine (flexion-extension, lateral tilt, rotation) via Numeric Rating Scale (NRS). In addition, they stated the subjective restriction of their general cervical mobility and for each plane in a five-point Likert scale from 0 (none) to 4 (complete) as Patient-Reported Restriction Score (PRRS) (Scholz et al., 2021; Whitcroft et al., 2010). This PRRS was also applied for the evaluation of the impairment of the following ADLs: personal hygiene, dressing, eating, drinking, sleeping, housework, overhead work, reading, leisure activities, walking/strolling, cycling, driving, conversation, professional activity. The Short-Form 8 (SF-8, German version) was used to assess the HR-QoL (Ellert et al., 2005). Physical component (PCS) and mental component summary (MCS) were analyzed separately (Ellert et al., 2005).

**Fig. 1:**
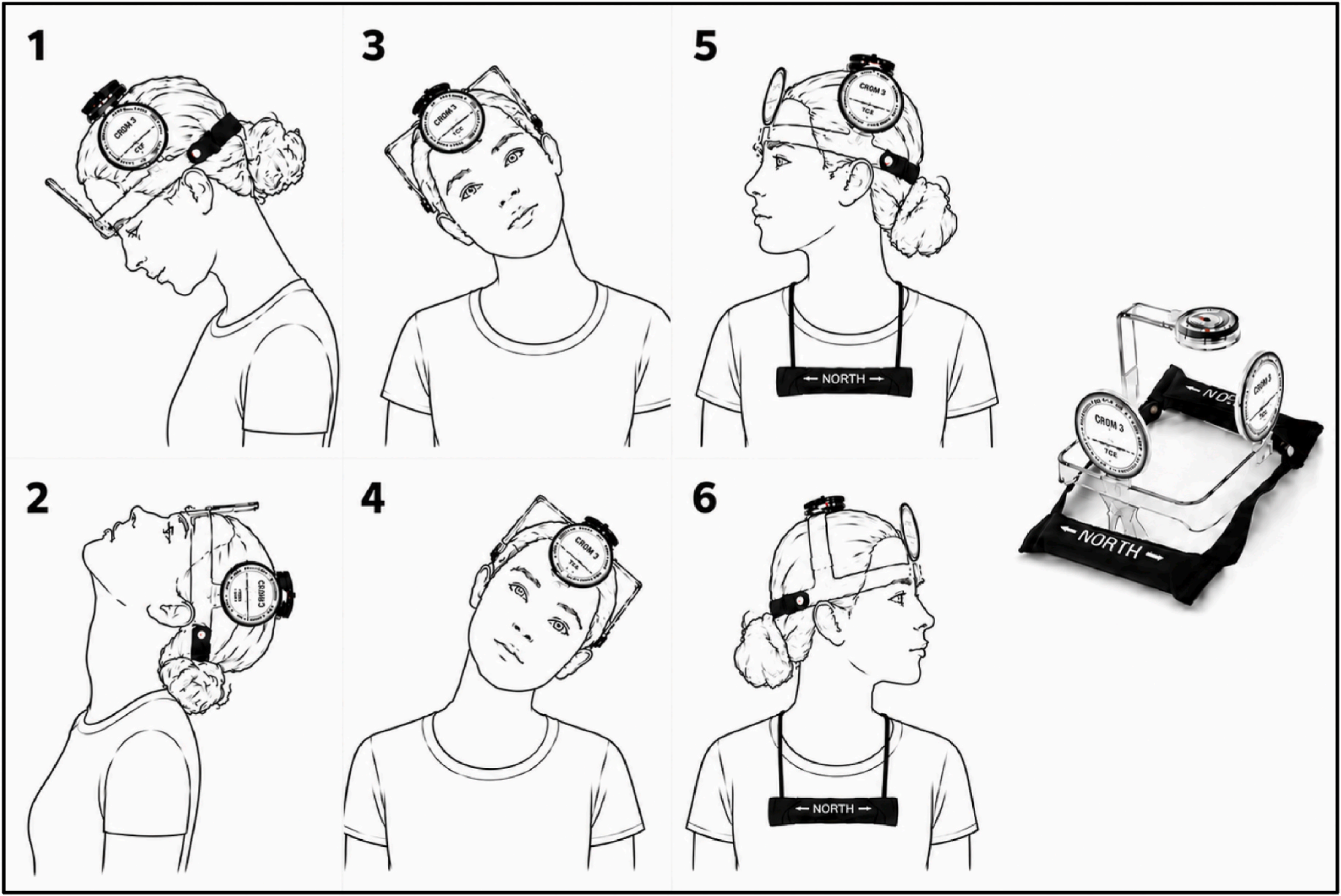
Measurement procedure with the CROM-3 goniometer (right side) showing maximum active head movement in each direction in all three planes of motion (1 – flexion, 2 – extension, 3 – right lateral tilt, 4 – left lateral tilt, 5 – right rotation, 6 – left rotation). The black magnetic necklace is required for the initial positioning of the measuring needle to evaluate the rotational movement with the goniometer directly above the head. The original photos showing the measurement process were turned into a sketch using ChatGPT 5.6 (OpenAI, San Francisco, California, USA).

After these subjective classifications of cervical mobility, the objective angular CROM was measured by *CROM-3* goniometer (Performance Attainment Associates, Roseville, Minnesota, USA) in each plane. Measurements were performed with the patient seated upright. After documentation of the resting position in sagittal and coronal plane, the active maximal movement in each direction was measured. The measurement process is depicted in Fig. 1. All angular measurements reported in this study are resting-position-corrected, representing the true range-of-motion from the individual neutral head position to maximal excursion. Composite scores were calculated as the sum of corrected individual directions: total flexion-extension, total lateral tilt, and total rotation.

All clinical data were collected directly the day before surgery (t_0_), 3months (t_1_) and 1 year after surgery (t_2_). Concerning the overall subjective effect of the surgical treatment, the Patient Satisfaction Index (PSI; modified subitem of the North American Spine Society outcome questionnaire) was applied (Scholz et al., 2021). To screen for the progress of bony fusion, the extent of trabecular bone bridges between the vertebral bodies was evaluated on CT scans at the 1-year follow-up and graded subjectively as “complete”, “partial” and “absent” bony fusion (Oshina et al., 2018).

Data analysis was done with Microsoft Excel 16 (Microsoft Cooperation, Redmond, Washington, USA) and SPSS Statistics 29 (IBM Cooperation, Armonk, New York, USA). Sankey plots were generated with Python 3.11 (SciPy 1.12). The three measurement timepoints were compared using Friedman's Two-Way Analysis of Variance by Ranks for related samples. Wilcoxon Signed-Rank test was applied for post-hoc pairwise comparisons and the adjusted significance level after Bonferroni correction was reported. For assessing normal distribution, Shapiro-Wilk test was used. Our data did not show a consistently normal or non-normal distribution. For the sake of clarity, we have provided the means and standard deviations throughout for the descriptive statistics. Linear regression analyses were performed to identify factors associated with the change in CROM goniometer measurements and PRRS from baseline to 1 year postoperative and to compare the predictive values of the individual factors sex, age, Body-Mass-Index (BMI), physical workload and pain intensity. A p-value <0.05 was used as significance limit.

## 3 Results

Overall, 85 patients receiving two-level ACDF could be included, whereas 16 patients had to be excluded from further analysis due to intraoperative plate fixation for instability (n = 1), prior cervical spine surgery (n = 2), or incomplete follow-up data (twelve patients lost to follow-up after 1 year and one patient lacking 3 months follow-up).

**Fig. 2:**
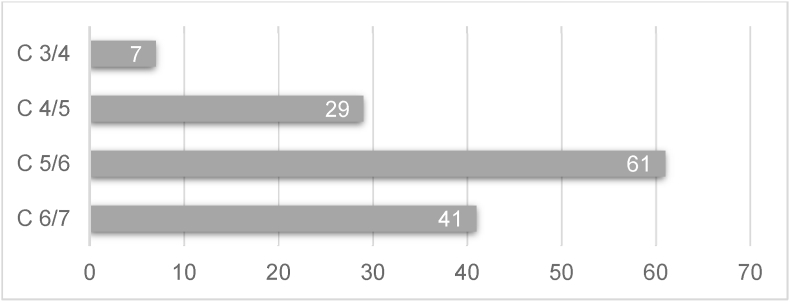
Distribution of the surgically addressed cervical levels.

This resulted in 69 complete patient datasets for evaluation with a mean age of 56.7 ± 11.0 years (range 34–85) and predominantly males (66.7% versus 33.3% females). The patients were in average in pre-obese status with a BMI of 27.5 ± 11.0 kg/m^2^ (range 19.1–46.1). Spinal decompression was performed due to DCM in 29% (n = 20) and for isolated radiculopathy in 39% (n = 27) of the cases. For the remaining 22 cases (31.9%), a combination of both was addressed. All patients received surgery for the levels C3 to C7 (distribution shown in Fig. 2). In 63 (91.3%) patients the addressed levels were right next to each other. This results in six patients where the surgery was done on distant levels, meaning simultaneously on C4/5 and C6/7 (n = 3), C3/4 and C5/6 (n = 2) and C3/4 and C6/7 (n = 1).

Concerning the fusion rates, we found 31 (44.9%) patients having complete bone bridges between the vertebral bodies of both segments, and 36 (52.2%) patients showing “partial” fusion of at least one treated segment but with new bony outgrowth extending beyond the vertebral bodies. There were two patients (2.9%) without substantial signs of bony outgrowth.

### 3.1 Clinical outcomes

Table 1 shows the pain and HR-QoL courses from preoperatively to follow-up at 3 months and 1 year. Neck pain was significantly reduced in average about −1.2 NRS points at rest (p = 0.007) and −1.5 NRS points under strain (p < 0.001) after 3 months. For the brachialgia, pain reduction was slightly higher with −2.0 points at rest (p < 0.001) and −2.9 points under strain (p < 0.001). There was no significant change of pain intensity from 3 months to 1 year postoperatively (range 0 - 0.4 NRS points; p = 0.291-1.000). The HR-QoL was already improved after 3 months, with an increase of +4.4 points in both components of the SF-8 (PCS and MCS, p < 0.001 each). This positive trend continued for 1 year after the operation (Table 1).

**Table 1:** Clinical outcome.

	Baseline t_0_	p t_0_-t_1_	3 months t_1_	p t_1_-t_2_	1 year t_2_	p t_0_-t_2_	Overall p*
NRS neck - at rest	2.9 ± 2.4	**0.007**	1.7 ± 1.8	1.000	1.7 ± 1.9	**0.010**	**<0.001**
NRS neck - under strain	4.8 ± 2.8	**<0.001**	3.3 ± 2.4	0.561	3.7 ± 3.0	**0.019**	**<0.001**
NRS arm - at rest	3.2 ± 2.8	**<0.001**	1.2 ± 1.7	0.291	1.5 ± 2.2	**0.004**	**<0.001**
NRS arm - under strain	5.0 ± 3.3	**<0.001**	2.1 ± 2.1	1.000	2.4 ± 2.6	**<0.001**	**<0.001**
SF-8: PCS	34.2 ± 9.2	**<0.001**	38.6 ± 8.4	0.409	42.8 ± 9.1	**<0.001**	**<0.001**
SF-8: MCS	39.9 ± 11.7	**<0.001**	44.3 ± 11.5	**0.022**	47.6 ± 10.6	**0.065**	**<0.001**

Pain scores via Numeric Rating Scale (NRS: range 0-10) and health-related quality of life via Short-form 8 (SF-8) with physical component summary (PCS) and mental component summary (MCS) at baseline (t_0_), after 3 months (t_1_) and 1 year (t_2_) as Mean ± SD. Highlighted are p-values <0.05. p* = p-values for the Friedman's Two-Way Analysis of Variance for all three measurements.

**Fig. 3:**
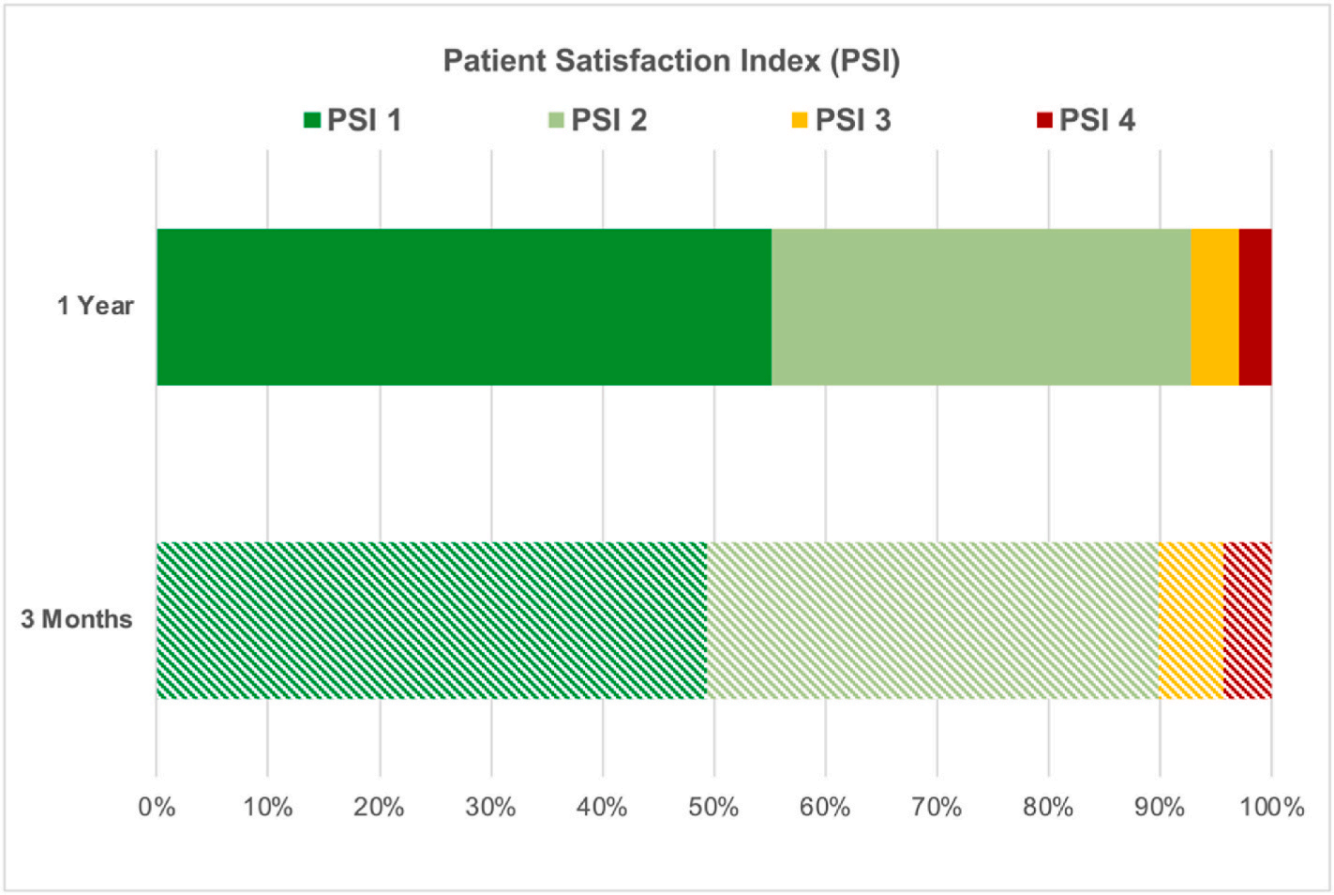
Percentage of all 69 patients for each Patient Satisfaction Index value (1 – completely satisfied – in dark green; 2 – satisfied, but residual complaints – in light green; 3 – symptoms improved, but not as much as expected – in yellow; 4 – symptoms unchanged or worsened – in red) and the two follow-up examinations: 3 months as diagonal-striped bar and 1 year as filled stacked bar.

The patient satisfaction with the surgery was high, showing 89.9% satisfied patients (PSI 1 + 2) after 3 months and 92.8% after 1 year. Of all treated patients, 2.9% had no effect or worsened 1 year after surgery (Fig. 3).

### 3.2 Objective cervical range-of-motion

Objective mobility in the different planes of cervical movement worsened 3 months postoperatively (t_0_-t_1_: flexion-extension −11.5°, p < 0.001; total lateral tilt −8.0°, p < 0.001; total rotation −3.6°, p = 0.062) with a significant recovery to the 1 year follow-up (t_1_-t_2_: flexion-extension +10.0°, p = 0.004; total lateral tilt +7.3°, p < 0.001; total rotation +6.5°, p = 0.009), resulting in a statistically unchanged CROM from pre-to 1 year postoperatively (t_0_-t_2_) with −1.5° flexion-extension (p = 0.700), −0.7° total lateral tilt (p = 0.700) and +2.9° for total rotation (p = 1.000; Table 2).

**Table 2:** Objective cervical range-of-motion (CROM).

	Baseline t_0_	P t_0_-t_1_	3 months t_1_	P t_1_-t_2_	1 year t_2_	P t_0_-t_2_	Overall p*
**Total flexion-extension**	90.4 ± 21.7	**<0.001**	78.9 ± 18.9	**0.004**	88.9 ± 16.5	0.700	**<0.001**
Flexion	49.7 ± 14.5	**<0.001**	41.7 ± 11.0	**0.011**	47.0 ± 11.6	0.136	**<0.001**
Extension	40.6 ± 11.4	0.058	37.1 ± 11.9	**0.017**	41.5 ± 10.8	1.000	**0.010**
**Total lateral tilt**	55.3 ± 14.9	**<0.001**	47.3 ± 15.7	**<0.001**	54.6 ± 16.1	0.700	**<0.001**
Right lateral tilt	27.8 ± 7.9	**<0.001**	23.5 ± 7.4	**0.006**	26.2 ± 9.0	0.444	**<0.001**
Left lateral tilt	27.5 ± 8.6	**<0.001**	23.8 ± 9.2	**0.001**	28.0 ± 9.2	1.000	**<0.001**
**Total rotation**	94.3 ± 20.6	0.062	90.7 ± 17.1	**0.009**	97.2 ± 19.2	1.000	**0.007**
Right rotation	46.6 ± 10.8	0.062	44.3 ± 8.9	**0.044**	47.4 ± 10.1	1.000	**0.018**
Left rotation	47.8 ± 11.4	**0.034**	46.4 ± 9.6	**0.018**	49.8 ± 10.6	1.000	**0.007**

Objective measurements of the *CROM-3* goniometer in degrees of all patients as mean values ± SD at baseline (t_0_), 3 months (t_1_) and 1 year (t_2_). Highlighted are p-values <0.05. p* = p-values for the Friedman's Two-Way Analysis of Variance for all three measurements.

**Fig. 4:**
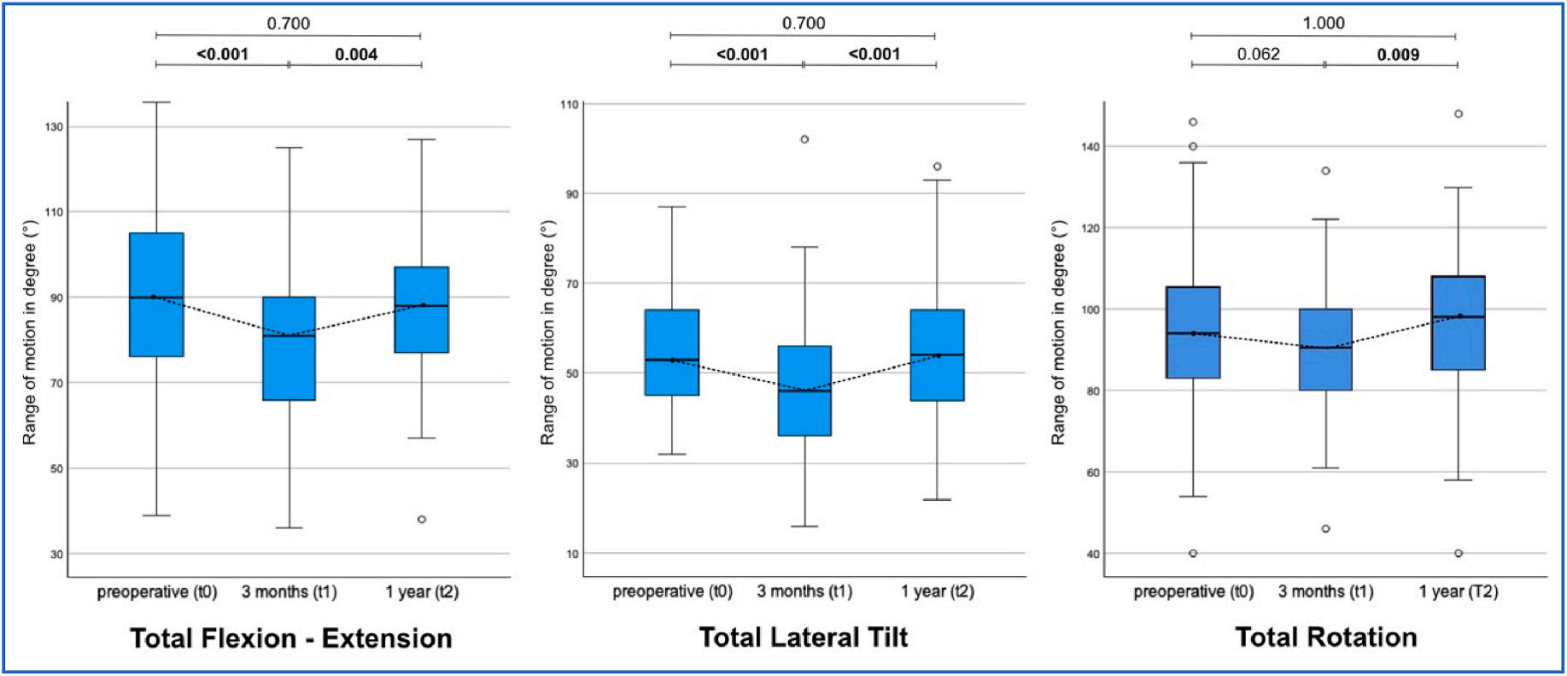
Box plots showing the total range-of-motion before surgery (t_0_), 3 months after surgery (t_1_), and 1 year after surgery (t_2_). Highlighted are p-values <0.05.

Fig. 4 additionally depicts the course for the composite total CROM in all three planes of movement.

### 3.3 Subjective cervical range-of-motion

**Fig. 5:**
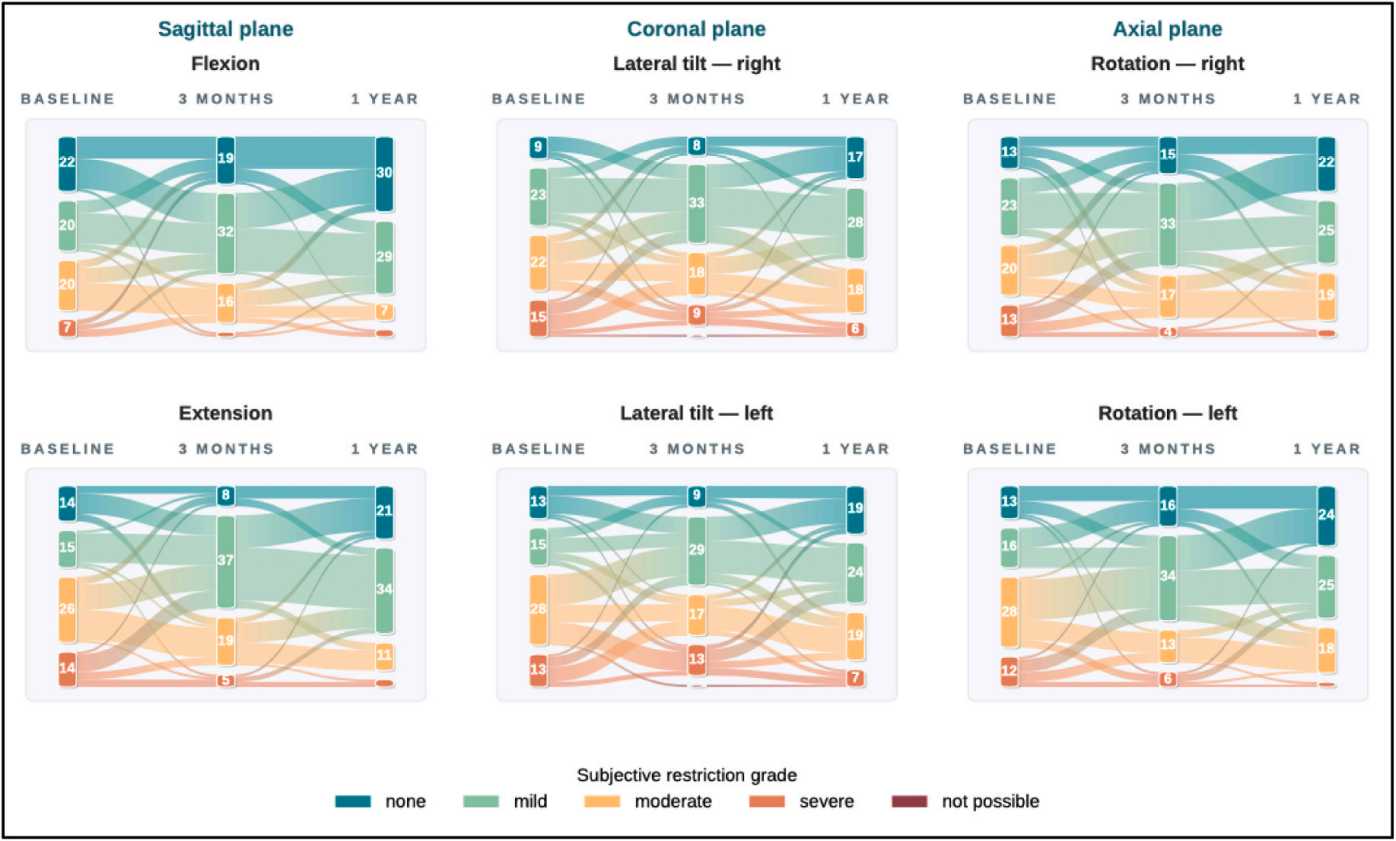
Sankey plots for the Patient-Reported Restriction Score (PRRS: dark green = “no restriction”; slight green = “mild”; yellow = “moderate”; red = “severe”; dark red = “no movement possible”) of all patients at baseline (t_0_ - left) and after 3 months (t_1_ - middle) and 1 year (t_2_ - right) for all planes of movement. The ribbon width is proportional to the number of patients moving between the restriction grades across the different timepoints and the color adapts from the source to the destination grades. The node numbers are patient counts per PPRS value. The Friedman's Two-Way Analysis of Variance for all three measurement timepoints revealed a significant shift for all movement planes (p < 0.001-0.024, Supplement 1).

General subjective CROM measured by PRRS, showed significant improvement from in average 2.0 ± 1.1 points to 1.3 ± 0.8 points after 1 year (p < 0.001). Also, for each single plane of movement, there was a significant improvement 1 year after surgery with a reduction of the mean PPRS at minimum of 0.4 points for flexion (p = 0.036) and right lateral tilt (p = 0.028) to a maximum of 0.7 points for the extension (p = 0.001). Only the left lateral tilt revealed no significant reduction (1.6 ± 1.0 to 1.2 ± 1.0, p = 0.111; Supplement 1). The change in PPRS categories from preoperatively to both follow-up timepoints is depicted in Fig. 5. There was a shift from mostly moderate (yellow) or severe (red) restricted patients prior to surgery to predominantly mild (light green) or not at all (dark green) restricted patients over time, up to the 1-year follow-up. This effect could be seen in all planes of movement.

The evaluated ADLs for the three timepoints showed an increase of the number of patients without or only mild impairment in their activities from prior to the surgery to 1 year postoperatively (Table 3). There was a higher number of patients avoiding some activities 3 months after surgery, especially cycling, driving and leisure activities. That effect disappeared after 1 year, with consequently more patients performing the evaluated activities compared to before the operation.

**Table 3:** Impairment of various activities of daily living (ADLs).

	t	Impairment due to a restriction in cervical spine mobility in %					n
		none	mild	moderate	severe	complete	
Personal hygiene	t_0_	**43.5**	29.0	21.7	5.8	0	69
	t_1_	**52.2**	30.4	11.6	5.8	0	69
	t_2_	**75.4**	20.3	4.3	0	0	69
Dressing	t_0_	**42.0**	34.8	15.9	7.2	0	69
	t_1_	**53.6**	31.9	8.7	5.8	0	69
	t_2_	**66.7**	30.4	1.4	1.4	0	69
Eating	t_0_	**62.3**	26.1	7.2	4.3	0	69
	t_1_	**66.7**	17.4	8.7	7.2	0	69
	t_2_	**84.1**	14.5	1.4	0	0	69
Drinking	t_0_	**66.7**	17.4	14.5	1.4	0	69
	t_1_	**69.6**	20.3	5.8	4.3	0	69
	t_2_	**82.6**	14.5	2.9	0	0	69
Sleeping	t_0_	21.7	23.2	**30.2**	23.2	1.4	69
	t_1_	33.3	**34.8**	21.7	10.1	0	69
	t_2_	**44.9**	31.9	15.9	7.2	0	69
Housework	t_0_	17.4	**29.0**	17.4	26.1	2.9	64
	t_1_	**30.4**	26.1	26.1	5.8	2.9	63
	t_2_	**47.8**	31.9	14.5	4.3	1.5	69
Overhead work	t_0_	11.6	13.0	17.4	**36.2**	17.4	66
	t_1_	7.2	17.4	**27.5**	15.9	13.0	56
	t_2_	17.4	**31.9**	20.3	21.7	5.8	67
Reading	t_0_	**39.1**	24.6	20.3	14.5	0	68
	t_1_	**55.1**	20.3	20.3	2.9	0	68
	t_2_	**65.2**	21.7	10.1	2.9	0	69
Leisure activities	t_0_	18.8	20.3	20.3	**21.7**	13.0	65
	t_1_	**24.6**	21.7	18.8	8.7	5.8	55
	t_2_	31.9	**33.3**	24.6	5.8	1.4	67
Walking/Strolling	t_0_	**39.1**	26.1	15.9	15.9	2.9	69
	t_1_	**49.3**	31.9	14.5	4.3	0	69
	t_2_	**68.1**	21.7	8.7	1.4	0	69
Cycling	t_0_	15.9	13.0	14.5	13.0	**17.4**	51
	t_1_	**14.5**	13.0	5.8	4.3	2.9	28
	t_2_	**31.9**	23.2	11.6	7.2	5.8	55
Driving	t_0_	24.6	**26.1**	24.6	14.5	2.9	64
	t_1_	23.2	**33.3**	11.6	2.9	4.3	52
	t_2_	**39.1**	37.7	11.6	5.8	1.4	66
Conversation	t_0_	**65.2**	17.4	14.5	2.9	0	69
	t_1_	**66.7**	18.8	14.5	0	0	69
	t_2_	**69.6**	21.7	5.8	2.9	0	69
Professional activity	t_0_	**20.3**	13.0	11.6	17.4	17.4	55
	t_1_	**11.6**	8.7	5.8	7.2	7.2	28
	t_2_	**27.5**	15.9	17.4	11.6	7.2	55

Impairment of various activities of daily living (ADLs) due to a restriction in cervical spine mobility at baseline (t_0_), after 3 months (t_1_) and 1 year (t_2_) as percentage values out of all 69 patients. Highlighted are the most frequently reported degrees of impairment. Right Column (n): number of patients performing the activity – the patients missing to the overall included number of 69 stated “I do not do this activity, or it does not concern me”.

### 3.4 Regression analysis

The regression analysis showed an advanced age to be an independent negative predictor for an increased objective CROM 1 year after surgery, whereas flexion-extension (β = −0.357, p = 0.007) and lateral flexion (β = −0.305, p = 0.023) revealed significant correlations and the rotation almost a statistical trend (β = −0.261, p = 0.055; Supplement 2). Additionally, neck pain at rest was a positive predictor for an increased rotation after 1 year (β = 0.575, p = 0.007) and arm pain under strain negatively for the subjective CROM (β = −0.422, p = 0.036).

## 4 Discussion

We evaluated 69 patients receiving two-level ACDF for a degenerative spinal disease concerning their objective and subjective CROM, HR-QoL and pain courses. We could show, that the subjective CROM was consistently improved and the objective measurements were stable without deterioration 1 year after surgery. Associated, neck and arm pain were significantly reduced and HR-QoL enhanced.

Spinal surgeries with associated bony fusion are expected to reduce spinal mobility. In case of healthy spinal segments, this assumption seems to be plausible, whereas in degenerated segments, the CROM is usually already restricted and associated pain leads to additional avoidance of movement. Since cervical fusion surgeries are common, this is a key point in everyday clinical practice and a usual concern of affected patients prior to operations. Therefore, precise information of the consequences of segmental fusion for overall spinal mobility will have direct impact on patient counseling and should be addressed more extensively.

For single-level ACDF, our group has previously demonstrated that the objective CROM is getting better within 1 year after surgery (Scholz et al., 2021). Other studies could show that the active CROM increases in all planes 6 months after surgery, independently of the number of levels fused (Landers et al., 2013). Focused on two-levels treated (n = 13), Landers et al. showed a significant increase of the pain-free CROM of +13.5° in sagittal, +13.0° in coronal and +12.4° in rotational plane (Landers et al., 2013). For two-level ACDF, our study represents the largest cohort within the recent literature. We could show significantly reduced degrees for all planes of objective CROM measurements after 3 months. At that point, the most average reduction was present in flexion-extension with −11.5° (p < 0.001). The lateral tilt showed a reduction of −8.0° (p < 0.001) and rotation of −3.6° (p = 0.062). However, in all three planes, a significant recovery occurred within the following months, leading to an overall marginally, non-significant affected CROM from pre-to 1 year postoperatively. The flexion-extension was slightly reduced with −1.5° (p = 0.700) like the lateral tilt with −0.7° (p = 0.700). The rotation was even increased with +2.9° (p = 1.000). So, we could not prove a reduced motion due to fusion surgery for the longer term, also in two-level ACDF. Nevertheless, the recovery period appears to be longer than following single-level ACDF, and the restrictions seem to be more pronounced, because one-level surgeries showed no significant reduction after 3 months and even improved CROM in all directions, while significant only for the rotation after 1 year (Scholz et al., 2021).

Independent from objective CROM measurements, the subjective mobility was significantly increased after two-level ACDF, comparable to previous single-level results. This stands in obvious contrast to unfounded rather emotional fears surrounding segmental fusion. The early postoperative course after 3 months indicated that recovery had already begun with slightly improved PRRS in all planes of movement (Fig. 5) and correspondingly for the ADLs (Table 3). But several activities, especially cycling, driving and sports, were rather avoided (see right row at timepoint t_1_ within Table 3). It therefore appears that patients' activity level remained limited after 3 months. However, after 1 year, there was a significant higher proportion of only slightly (49.3%) or unrestricted (13.0%) patients for general cervical mobility. The average PPRS was reduced by 0.7 points (p < 0.001) in general and by 0.4-0.7 points for the different planes of motion (p = 0.001-0.111). So, the initial limitations diminish over time and patients were more mobile 1 year after surgery than before the operation. This is also represented by the higher proportion of only mild or without subjective restrictions for all planes of movement at the follow-up timepoints compared to preoperatively, as is evident from the Sankey plots in Fig. 5. Accordingly, all ADLs showed fewer limitations after 1 year. Out of all evaluated ADLs, overhead work showed the highest amount of restriction with 47.8% feeling moderately to severely restricted even after 1 year. This is consistent with previous studies and seems to be due to the most pronounced motion loss in the sagittal plane (Scholz et al., 2021).

The most likely reason for the increased subjective cervical mobility seems to be the significantly reduced pain intensity, so that the adjunct levels can compensate the fixation of the addressed levels (Langenfeld et al., 2018; Riffitts et al., 2023; Kasliwal et al., 2016; Landers et al., 2013). Neck pain is directly associated with restricted CROM (Stenneberg et al., 2017). Within our evaluation, NRS was significantly reduced at the neck and arm at rest as well as under strain for both follow-up timepoints. The extent of pain reduction was slightly lower than in our previous single-level cohort, whereas the average difference was 0.6 NRS points for the neck (rest and strain), as well as for radiating pain at rest. The difference for arm pain under strain was only 0.2 NRS points. This slightly lower pain reduction with two-level ACDF could be an additional factor for the inferior mobility compared to the single-level cohort.

We want to emphasize the importance of such subjective movement limitations, HR-QoL and ADLs as PROMs, rather than focusing solely on mobility changes measured by goniometers or radiographs. Those subjective perceived restrictions are underrepresented yet. Additionally, the significant increase of both SF-8 components through the two-level ACDF means a very effective surgical procedure for HR-QoL, which is also reflected by the high number of 92.8% satisfied patients (PSI 1 + 2).

The regression analysis confirmed, matching to the single-level data, that the patients’ age significantly influences postoperative mobility changes (Scholz et al., 2021). So, younger patients have greater potential to improve their spinal mobility, likely due to higher compensatory capacity of adjacent, non-fused and mostly non-degenerative segments.

The advantage of this study is the homogeneous cohort that is highly comparable to previously published data on single-level ACDF and data quality is high due to prospective sampling. Nevertheless, there are some relevant limitations. Incomplete datasets were excluded (n = 13), which leads to some uncertainty. We excluded one patient receiving a cervical plate, because this reflects a different indication. For those patients, preoperative pain scores as well as the cervical spine mobility are expected to be different based on the underlying instability, and also the postoperative course could be affected through the plate itself and its’ implantation, which is a slightly more extensive surgical procedure. This effect has to be clarified through further specialized trials. Another limitation is that we did not evaluate the overall degeneration of the cervical spine regardless of the surgically treated levels, whereas this could be an influencing factor for spinal mobility. There are no valuable data on the relationship between additional segmental degeneration and CROM restriction, the same applies to the postoperative fusion status. The progress of bony fusion at our 1-year follow-up evaluated by CT scans were mostly complete (44.9%) or at least advanced (52.2%), whereas no substantial association to pain course and CROM could be found, most likely due to the too small patient count and the basically unknown impact of the various stages of bony fusion on the general mobility of the cervical spine. Of the two patients without substantial bone growth through the surgical addressed levels, one suffered from persistent severe pain, whilst the other was satisfied with almost complete pain relief. This highlights the heterogeneity within this nevertheless relevant topic of fusion rates and pseudarthrosis, which requires further investigation.

Additionally, we could not determine whether the effects on the CROM were primarily due to pain reduction or biomechanical changes. Also, the clear impact of cervical mobility on the different ADLs could not be clarified, whereas some evaluations postulate only small relevance for the long-term because of accommodation (Langenfeld et al., 2018). So, the mobility effect on ADLs and HR-QoL may be overestimated, making dedicated studies necessary to draw pathophysiological conclusions (Riffitts et al., 2023). The goniometric CROM measurements were carried out by a trained study group of five surgeons, which possibly leads to a certain degree of inter-observer heterogeneity. However, the intra- and inter-observer reliability for the CROM goniometer is described in the literature as good to excellent (Audette et al., 2010; Florêncio et al., 2010).

Our evaluation addresses a small, but clearly defined cohort, whereas further surgical procedures should be evaluated in the future concerning their PROMs, like multi-level ACDF or posterior foraminal decompressions. Patients with three or more levels fused showed reduced CROM in prior evaluations, whereas the consequences for the subjective restrictions are not well described (Landers et al., 2013). Furthermore, additional long-term observations are needed to gain insights into how the effects observed after 1 year will evolve and what consequences this will have for the adjacent segments.

## 5 Conclusions

After two-level ACDF due to degenerative cervical spine disease, the objective cervical range-of-motion was temporarily reduced with complete recovery 1 year after surgery. The subjective cervical mobility as well as the health-related quality of life and associated activities of daily living were already improved 3 months postoperatively, most likely due to the significant reduction of arm and neck pain. This resulted in high patient satisfaction with the two-level procedure. Based on this evaluation, concerns about potential postoperative mobility limitations can be actively addressed.

## Declaration of competing interest

The authors declare that they have no known competing financial interests or personal relationships that could have appeared to influence the work reported in this paper.
