# Supplementary material for: Subjective and objective cervical spine mobility after two-level anterior cervical discectomy and fusion - A prospective observational trial
Source: Brain Spine. 2026 Jul 30;6:106250. doi: 10.1016/j.bas.2026.106250 (PMC13452230; doi:10.1016/j.bas.2026.106250)
Supplement: Supplementary file 1 [file mmc1.docx]

| **SUPPLEMENT 1:** Patient-Reported Restriction Score (PRRS) | | | | | | | |  |
| --- | --- | --- | --- | --- | --- | --- | --- | --- |
|  | | Baseline  t_0_ | p  t_0_-t_1_ | 3 months  t_1_ | p  t_1_-t_2_ | 1 year  t_2_ | p  t_0_-t_2_ | Overall p* |
| General | Mean ± SD | 2.0 ± 1.1 | 0.317 | 1.7 ± 0.9 | 0.072 | 1.3 ± 0.8 | **<0.001** | **<0.001** |
|  | 0 | 11.6 % |  | 10.1 % |  | 13.0 % |  |  |
|  | 1 | 15.9 % |  | 24.6 % |  | **49.3 %** |  |  |
|  | 2 | **37.7 %** |  | **52.2 %** |  | 30.4 % |  |  |
|  | 3 | 26.1 % |  | 11.6 % |  | 5.8 % |  |  |
|  | 4 | 8.7 % |  | 1.4 % |  | 1.4 % |  |  |
| Flexion | Mean ± SD | 1.2 ± 1.0 | 1.000 | 1.0 ± 0.8 | 0.151 | 0.8 ± 0.8 | **0.036** | **0.003** |
|  | 0 | 31.9 % |  | 27.5 % |  | **43.5 %** |  |  |
|  | 1 | **39.0 %** |  | **46.4 %** |  | 42.0 % |  |  |
|  | 2 | 29.0 % |  | 23.2 % |  | 10.1 % |  |  |
|  | 3 | 10.1 % |  | 2.9 % |  | 4.3 % |  |  |
|  | 4 | 0 % |  | 0 % |  | 0 % |  |  |
| Extension | Mean ± SD | 1.6 ± 1.0 | 0.605 | 1.3 ± 0.8 | 0.065 | 0.9 ± 0.8 | **0.001** | **<0.001** |
|  | 0 | 20.3 % |  | 11.6 % |  | 30.4 % |  |  |
|  | 1 | 21.7 % |  | **53.6 %** |  | **49.3 %** |  |  |
|  | 2 | **37.7 %** |  | 27.5 % |  | 15.9 % |  |  |
|  | 3 | 20.3 % |  | 7.2 % |  | 4.3 % |  |  |
|  | 4 | 0 % |  | 0 % |  | 0.0 % |  |  |
| Right lateral tilt | Mean ± SD | 1.6 ± 1.0 | 0.983 | 1.5 ± 0.9 | 0.317 | 1.2 ± 0.9 | **0.028** | **0.006** |
|  | 0 | 13.0 % |  | 11.6 % |  | 24.6 % |  |  |
|  | 1 | **33.3 %** |  | **47.8 %** |  | **40.6 %** |  |  |
|  | 2 | 31.9 % |  | 26.1 % |  | 26.1 % |  |  |
|  | 3 | 21.7 % |  | 13.0 % |  | 8.7 % |  |  |
|  | 4 | 0 % |  | 1.4 % |  | 0 % |  |  |
| Left lateral tilt | Mean ± SD | 1.6 ± 1.0 | 1.000 | 1.5 ± 1.0 | 0.243 | 1.2 ± 1.0 | 0.111 | **0.024** |
|  | 0 | 18.8 % |  | 13.0 % |  | 27.5 % |  |  |
|  | 1 | 21.7 % |  | **42.0 %** |  | **34.8 %** |  |  |
|  | 2 | **40.6 %** |  | 24.6 % |  | 27.5 % |  |  |
|  | 3 | 18.8 % |  | 18.8 % |  | 10.1 % |  |  |
|  | 4 | 0 % |  | 1.4 % |  | 0 % |  |  |
| Right rotation | Mean ± SD | 1.5 ± 1.0 | 0.166 | 1.1 ± 0.8 | 1.000 | 1.0 ± 0.9 | **0.015** | **0.002** |
|  | 0 | 18.8 % |  | 21.7 % |  | 31.9 % |  |  |
|  | 1 | **33.3 %** |  | **47.8 %** |  | **36.2 %** |  |  |
|  | 2 | 29.0 % |  | 24.6 % |  | 27.5 % |  |  |
|  | 3 | 18.8 % |  | 5.8 % |  | 4.3 % |  |  |
|  | 4 | 0 % |  | 0 % |  | 0 % |  |  |
| Left rotation | Mean ± SD | 1.6 ± 1.0 | **0.010** | 1.1 ± 0.9 | 1.000 | 1.0 ± 0.9 | **0.001** | **<0.001** |
|  | 0 | 18.8 % |  | 23.2 % |  | 34.8 % |  |  |
|  | 1 | 23.2 % |  | **49.3 %** |  | **36.2 %** |  |  |
|  | 2 | **40.6 %** |  | 18.8 % |  | 26.1 % |  |  |
|  | 3 | 17.4 % |  | 8.7 % |  | 2.9 % |  |  |
|  | 4 | 0 % |  | 0 % |  | 0 % |  |  |
| Results of the Patient-Reported Restriction Score (PRRS: 0 = none; 1 = mild; 2 = moderate; 3 = severe; 4 = complete restriction) of all patients at baseline (t_0_) and after 3 months (t_1_) and 1 year (t_2_) as percentage and as Mean ± SD. Highlighted are the most frequently reported degrees of restriction and p-values <0.05. p* = p-values for the Friedman's Two-Way Analysis of Variance for all three measurements. | | | | | | | | |
