# Supplementary material for: Subjective and objective cervical spine mobility after two-level anterior cervical discectomy and fusion - A prospective observational trial
Source: Brain Spine. 2026 Jul 30;6:106250. doi: 10.1016/j.bas.2026.106250 (PMC13452230; doi:10.1016/j.bas.2026.106250)
Supplement: Supplementary file 2 [file mmc2.docx]

| **Supplement 2:** Regression analysis | | | | | | | | |
| --- | --- | --- | --- | --- | --- | --- | --- | --- |
|  | Total  flexion-extension | | Total  lateral tilt | | Total  rotation | | PRRS  (general) | |
|  | β | p | β | p | β | p | β | p |
| Gender | -0.006 | 0.964 | 0.083 | 0.532 | -0.004 | 0.975 | -0.086 | 0.469 |
| Age | -0.357 | **0.007** | -0.305 | **0.023** | -0.261 | 0.055 | 0.156 | 0.188 |
| Body mass index | -0.086 | 0.499 | 0.141 | 0.277 | 0.014 | 0.911 | 0.007 | 0.952 |
| Physical workload | 0.025 | 0.839 | -0.033 | 0.789 | -0.059 | 0.633 | 0.007 | 0.952 |
| Neck pain at rest | 0.159 | 0.441 | -0.057 | 0.786 | 0.575 | **0.007** | -0.043 | 0.822 |
| Neck pain under strain | 0.050 | 0.797 | 0.261 | 0.195 | -0.059 | 0.768 | -0.285 | 0.114 |
| Arm pain at rest | -0.227 | 0.331 | -0.176 | 0.461 | -0.461 | 0.054 | 0.094 | 0.660 |
| Arm pain under strain | 0.305 | 0.160 | 0.202 | 0.359 | 0.106 | 0.631 | -0.422 | **0.036** |
| Regression analysis of 69 patients to predict the differences in *CROM-3* goniometer measurements and patient-reported restriction scores (PRRS) for general restriction between baseline (t_0_) and at 1 year postoperatively (t_2_) from demographic variables and baseline pain scores. The standardized regression coefficient beta (β) is stated for each predictor. Highlighted are p-values <0.05. | | | | | | | | |
